# RS Content and eGI Value of Cooked Noodles (I): Effect of Cooking Methods

**DOI:** 10.3390/foods9030328

**Published:** 2020-03-11

**Authors:** Yu Tian, Ming Li, Aoxing Tang, Jay-Lin Jane, Sushil Dhital, Boli Guo

**Affiliations:** 1 Institute of Food Science and Technology, CAAS/Key Laboratory of Agro-Products Processing, Ministry of Agriculture and Rural Affairs, Beijing 100193, China; ytiancaas@163.com (Y.T.); tangaoxinghn@163.com (A.T.); 2 Department of Food Science and Human Nutrition, Iowa State University, Ames, IA 50011, USA; jjane@iastate.edu; 3 Department of Chemical Engineering, Monash University, Clayton, VIC 3800, Australia; sushil.dhital@monash.edu

**Keywords:** noodles, resistant starch, cooking methods, in vitro digestion

## Abstract

Noodles are widely consumed in China, which can be cooked in different ways. The effects of different cooking methods (boiling, steaming, microwave heating, stir-frying and frying) on the resistance starch (RS) content and digestive properties (digestion rate, digestibility and estimated glycemic index (eGI) value) of noodles were investigated. The RS content was greatly affected by the cooking time, and it was varied when the noodles were optimally cooked using different cooking methods. The RS contents of the microwaved and stir-fried noodles were relatively high (0.59%–0.99%), but it was lower (0.43%–0.44%) in the boiled and steamed noodles. Microwaved noodles showed the slowest digestion rate and the lowest eGI. Due to the limited water within fried noodles, none RS was found in the fried noodles, whereas stir-fried noodles showed RS_5_ formation from the XRD and DSC results. Compared with boiled and steamed noodles, the microwaved noodles showed a more compact morphology without porous holes on the surface, whereas fried noodles showed irregular morphology. The results indicated that the digestive properties of noodles made with the same ingredients can be greatly altered by using different cooking methods, and the digestive properties of different cooked noodles are worthy of confirmation using in vivo analysis.

## 1. Introduction

Noodles are staple foods for many Asian countries [1]. In China, noodles are cooked in a variety of ways, such as steaming, boiling, stir-frying, frying and simmering, which enable the noodles to have different tastes and flavors.

Starch is the major ingredient of noodles, which can be classified as rapidly digestive starch (RDS), slowly digested starch (SDS) and resistant starch (RS) [2] based on the digestion rate and extent in the human digestive tract. RS is the starch that cannot be absorbed in the small intestine of healthy individuals [3], but it can be utilized by microorganisms in the colon [4]. Studies have shown that RS can delay the increase of postprandial blood glucose, reduce body weight, promote insulin secretion and increase insulin sensitivity, playing an important role in preventing the occurrence and development of diabetes [5]. Further, RS can be divided into 5 groups [6]. RS_1_ is the starch that is physically inaccessible to digestion by entrapment in a non-digestible matrix. RS_2_ is composed of native starch granules of selected botanical sources. RS_3_ is retrograded starch and is created when gelatinization is followed by a retrogradation process. RS_4_, which is formed by chemical modification. RS_5_ is the amylose complexed with lipid, where the helical structure is hardly digested by the enzyme. However, RS, is not a ‘property’ of starch, but is a kinetic parameter largely determined by access of enzyme to the substrate (binding) and subsequent enzyme action (catalysis) as discussed elsewhere [7].

Compared with raw starch, the RS content of cooked starchy food is significantly reduced, and further influenced by different cooking methods. For example, the RS content of potato chips has been reported to increase after roasting, baking and shallow frying (frying in limited oil), whereas it decreases after steaming and frying [8]. After cooking, starch is mostly gelatinized [9], and amylase can hydrolyze the gelatinized starch more easily compared to raw or partially cooked starch in foods. Apart from starch gelatinization, cooking methods [10] can destroy the existing structure or develop the new structure (e.g., due to denaturation of protein and solubilization of fiber), that can affect both the rate [11] and extent of digestion of starch. The changes can be described as macro-level structure (food structure) as in micro-level and molecular structure (micro-level) [1,12] Compared with bread or puffed products, the dense packing of foods (pasta as an example) during the processing leads to reduced enzyme susceptibility [13]. Besides the digestion properties, the glycemic index (GI) value is an integrated index reflecting the postprandial blood glucose concentrations after food digestion, which is often used to classify the carbohydrate-rich foods [14]. However, GI evaluation in humans is labor-intensive and time-consuming, thus in vitro methods have been developed for estimating the GI of foods [15].

Noticeably, noodles are considered as a high glycemic index (GI) food [16] as the starch in boiled noodles, which is mostly gelatinized [9], can be easily hydrolyzed. Several approaches have been taken to reduce the glycemic response of noodles. It is found that noodles made from Tartary buckwheat flour could reduce the release of reducing sugar with an in vitro starch digestion and the 45% addition of Tartary buckwheat in noodles could benefit human health [17]. Apart from the starch, other ingredients in noodles, such as protein content [9] or the binding agent [18], can also affect noodle digestion. However, whether cooking methods can change the RS content of noodles, and if there is any correlation between the digestion rate and RS content of noodles cooked using different methods are not fully understood. 

Therefore, the objective of this study is to investigate the effects of different cooking methods (boiling, steaming, microwave heating, stir-frying and frying) on the RS content and digestive properties (digestion rate, digestibility and estimated glycemic index (eGI) value of noodles. The thermal properties, crystalline structure, and morphology of cooked noodles were explored using differential scanning calorimetry (DSC), X-Ray diffraction (XRD) and scanning electron microscope (SEM), respectively, to understand how the micro-structure and macro-structure affect the digestive properties of noodles using different cooking methods.

## 2. Materials and Methods

### 2.1. Materials

Wheat flour of Ningchun 4, a gift of Northwest Agricultural and Forestry University, was prepared using a laboratory Buhler mill (MLU 202, BUHLER, Switzerland) and passed through an 80-mesh sieve. Soybean oil (Arawana, Wilwar Trading Pte. Ltd, Beijing, China) was purchased from a local grocery store.

Resistant starch (RS) assay kit (Megazyme, Bray, Ireland) was used to measure the RS content of noodles prepared using different cooking methods and storage conditions. Pancreatin (Sigma, P7545 visceral, 8u/mg), Amyloglucosidase (AMG) (Sigma, A7096, Sigma-Aldrich, America), and Acetate buffer (pH 6.0, contains 4 M of CaCl_2_ and 0.49 M of MgCl_2_) were used for the in vitro digestion experiments. All of the chemicals used were of analytical grade.

### 2.2. Preparation of Noodles

White-salted noodles were prepared following the method of Tang et al. [19]. 100 g wheat flour, 1 g salt (1% based on flour weight), and distilled water (31.5% based on flour dry weight) were mixed using a mixer (JHMZ 200, Beijing Dongfu Jiuheng Instrument, Beijing, China) for 4 min. The dough was then rested for 30 minutes, and a noodle machine (JMTD-168/140,Beijing Dongfu Jiuheng Instrument, China ) was used to sheet the noodle dough, and the noodle strands were cut with a width of 2.0 mm and thickness of 1.0 mm. 

### 2.3. Preparation of Cooked Noodles 

Referring to the daily cooking methods of Chinese families, five cooking methods of boiling, steaming, stir-frying, frying and microwave were selected to cook the noodles. Using cooking time as a variable, the noodles cooked at the optimum cooking time (OCT) and the time before and after OCT were prepared. This will capture the eating habit of consumer that prefer the firm (al dente) or softer noodles. OCT was obtained through taking out a noodle string every 30 s and squeezed by a pair of transparent glass plates to observe the presence of the white core. The time when the white core disappeared was considered as the OCT for the noodles cooked with different methods [9]. 

Boiling: Noodles (20 g) were placed in 200 mL boiling water and boiled for 120, 150, 180, 210, and 240 s, with 210 s the OCT.

Steaming: Noodles (20g) were spread out evenly in trays with a thickness of about 2mm and steamed at 100 °C for 600, 900, 1200, and 1800 s, with 900 s the OCT.

Stir-frying: Raw noodles tend to stick together when stir-fried directly, thus noodles were partially gelatinized before stir-frying. Noodles (20 g) were boiled in 200 mL boiling water for 120 s and then cooled in running water for 30s. The boiled noodles were stir-fried in a pan (180 °C) with 5, 10, and 15 mL soybean oil for 30 and 120 s. Among them, 10 mL of oil stir-fried for 30 s is the optimum cooking condition.

Frying: Noodles (20 g) were fried in soybean oil (400 mL) in an electric frying pan (IESKIMOS, China) at 140, 160, 180, and 200 °C for 30 s and 120 s. Among them, fried at 180 °C for 30 s is the optimum cooking condition.

Microwave-cooking: Noodles (20 g) were put in a glass bowl with 200 mL boiling water and microwaved for 60, 120, 150, 180, and 240 s in a microwave oven (700 W, Midea, China) (on high) with 150 s the OCT.

The noodles cooked at different conditions were frozen immediately in liquid nitrogen. The samples were lyophilized, ground (MLX-400, RETSCH) and passed through a 100-mesh sieve for further analysis.

### 2.4. The Moisture Content of Cooked Noodles

Moisture contents (MC) of noodles cooked using different methods were determined according to AACC method 44-15A. The analysis was performed in triplicate. Freshly cooked noodles (Section 2.3, only the noodles cooked under OCT) were rested at room temperature for 5 min to equilibrate the moisture as well as drain the water before determining the moisture content. The moisture content was measured by heating the sample for 1 h at 130 °C in an oven, which was removed to a desiccator. The samples were weighed after they reached room temperature. Replicates were performed on the moisture content measurement.

(1) $$\mathrm{Moisture}\;\mathrm{content}(g/100g)=\frac{A}{B}\times 100,$$

*A* is the moisture loss in grams, and *B* is the original weight of the sample.

### 2.5. Wide-Angle X-ray Diffraction Patterns (XRD)

A D8 Advance X-ray diffractometer (Bruker, Madison, WI, USA) was used with Cu K_α_ radiation at 40 kV and 30 mA. Before analysis, dried noodle power was equilibrated in a sealed desiccator (with relative humidity of 55%) at room temperature for 12 h. The diffractogram scan was run between 2° and 40° (2*θ*) with a step size of 0.02° and at a rate of 5°/min. The relative crystallinity was calculated from the ratio of the crystalline area to the total diffractogram area using the methods of Li et al. [20] using a PeakFit software (Version 4.12 Systat Software, Inc., San Jose, CA, USA).

### 2.6. Differential Scanning Calorimetry (DSC)

The thermal properties of freshly cooked noodles (cooking under OCT) were analyzed in duplicate using a DSC (DSC 1, METTLER TOLEDO, USA) based on the method described by Teng et al. [21]. For the measurement, samples in excess water (three times the weight of starch) were equilibrated for 1 h at room temperature and heated from 0 to 140 °C using a high-pressure pan at a rate of 10 °C/min. The peak temperature (*Tp*) and retrogradation enthalpy change (∆H_R_) or gelatinization enthalpy change (∆H_G_) were determined from each endotherm using a built-in software (STARe system, Mettler Toledo, USA). 

### 2.7. Scanning Electron Microscope (SEM)

Noodles cooked using different methods were freeze-dried by ALPHA 1-2 LD plus freeze dryer (CHRIST, Germany). Noodle strands with a length of about 5 mm were fixed on the sample stage, which was sprayed with gold using JFC-1600 ion sputter (JEOL, Japan). The images were taken under a JSM-6510 LV scanning electron microscope (JEOL, Japan) at a magnification of 1500 times.

### 2.8. Determination of RS Content

The RS content of cooked noodles was determined using a K-RSTAR 06/18 kit from Megazyme (Bray, Ireland), according to the AOAC Method 2002.02 [22]. The samples (100 mg, accurately weighed) were incubated with pancreatic α-amylase and amyloglucosidase (AMG) (K-RSTAR 06/18; Megazyme, Bray, Ireland) for 16 h at 37 °C. The RS content was calculated based on the portion of the starch that was not hydrolyzed. All analyses were performed in duplicate for each sample, and results were reported as mean values.

### 2.9. In Vitro Starch Digestibility

In vitro starch digestibility was analyzed following the method of Wang [23] with slight modification. Freeze-dried noodle samples (cooking under OCT) (containing 50 mg starch, dsb) and water (2 mL) were added into 50 mL polypropylene centrifuge tubes. (The starch content of noodles was determined using a K-TSTA 04/2009 kit from Megazyme (Bray, Ireland)). After equilibrating at 37.0 °C for 20 min, 7.5 mL acetate buffer (pH 6.0) and 0.5 mL of porcine pancreatic α-amylase/amyloglucosidase mixture were added to the tubes. The noodle was digested in a water bath at 37 °C with stirring at 300 rpm, and the digesta (0.1 mL) were collected at 0, 10, 20, 30, 60, 90, 120, 150, 180, 210, 240 and 300 min. The digestion was stopped by adding 0.5 mL of ethanol (99% *v*/*v*), followed by centrifuging at 4000× *g* for 5 min. The hydrolyzed glucose content was measured using GOPOD reagent.

The digestograms were fitted to first-order kinetics [24,25] as shown in Equation (2):

(2) $$C_{t}=C_{\infty }\left(1-e^{-kt}\right),$$

where C_t_ is the concentration of product at a given time (t), $C_{\infty }$ is the concentration of product at the end of the reaction, and k is the first-order rate constant. For ease of interpretation, *C*_t_ may be expressed as the amount of starch digested as a percentage of the total starch content of the sample. A Logarithm of Slope (LOS) plot was obtained by expressing the first derivative of the first-order equation in logarithmic form (Equation (3)).

(3) $$\ln\left(\frac{dC}{dt}\right)=-\mathrm{kt}+\ln\left(C_{\infty }k\right),$$

where ln(dC/dt) represents the logarithm of the slope, and the equation describes a linear relationship between LOS and time of amylolysis, t. The resulting *k* and $C_{\infty }$ were used to construct model-fit starch digestion curves according to Equation.

### 2.10. Calculation of the Estimated Glycemic Index (eGI)

To calculate the estimated GIs of the samples, the areas under the digestograms (*AUC_exp_*) were calculated with Equation (4):

(4) $$AUC_{exp}=\left[C_{\infty }t+\frac{C_{\infty -0}}{K}\exp\left(-Kt\right)\right]\begin{array}{c} t2\\ t1 \end{array},$$

The hydrolysis index (HI) of each sample was calculated by dividing the area under its digestogram by the area under the digestogram of fresh white bread [13], as shown in Equation (5):

(5) $$HI=\frac{\mathrm{AUC}\;\mathrm{of}\;\mathrm{product}}{\mathrm{AUC}\;\mathrm{of}\;\mathrm{white}\;\mathrm{bread}}\times 100,$$

From the hydrolysis index obtained, the estimated glycemic index (eGI) (bread = 100) was calculated using the equation established by Goni et al. [13]: eGI = 39.7 + 0.548HI

## 3. Results 

### 3.1. Effects of Cooking Conditions on the RS Content of Noodles

**Figure 1:**
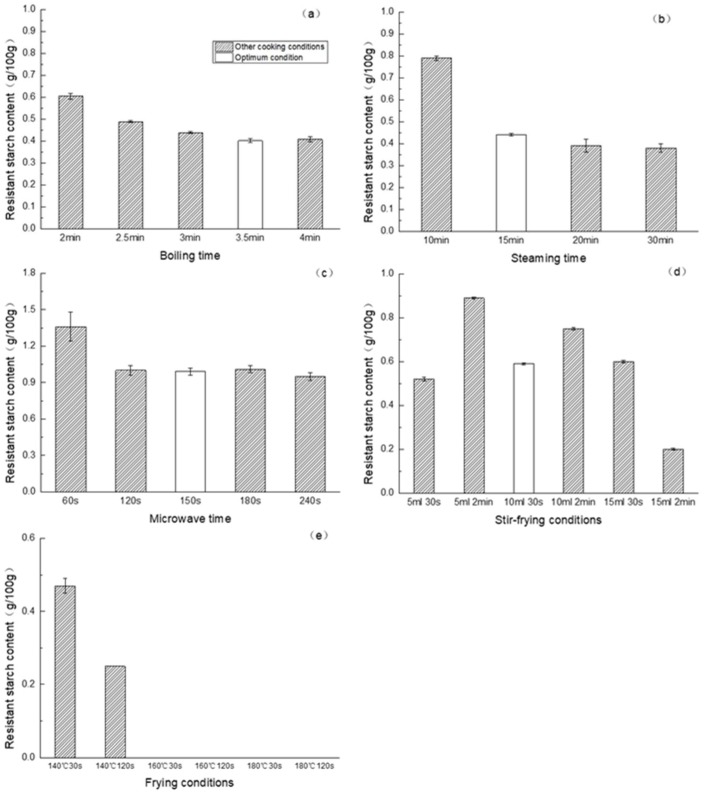
Effect of cooking conditions on resistant starch (RS) content of noodles (**a**) with different boiling times; (**b**) with different steaming times; (**c**) with different microwave-heating times; (**d**) with different stir-frying conditions; (**e**) with different frying conditions).

The RS content of raw noodles was 13.83 g/100 g, but it decreased significantly after cooking. After boiling, steaming and microwave cooking, the RS content decreased significantly with the cooking time, whereas the RS content did not change further after the noodles were cooked for the optimal cooking time (Figure 1a–c; Table S1–S3). The decrease in the RS content with longer cooking time could be explained by the higher degree of gelatinization of the starch within noodles, as the starch gelatinization peak (69.61 °C, Table 1) from all noodles under the optimum conditions were lost. Wang et al. [26] found that the hydrolysis percentage of cooked wheat starch increased to 37%, 56%, 66% and 73% for starches when the gelatinization degree was 14%, 37%, 71% and 85%, respectively.

The temperature, the amount of oil used and the cooking time varied during the stir-frying and frying process. The RS content of stir-fried noodles was significantly changed with the amount of oil used (Figure 1d, Table S4). When the amount of oil increased from 5 to 10 mL, the RS content of the noodles increased from 0.52 to 0.59 g/100 g. However, it did not change significantly when the amount of oil further increased. When the amount of oil was low (5 and 10 mL), the RS content of the stir-fried noodles increased with the cooking time. More starch-lipid complexes may form with prolonged contact time with oil. Yang [27] also found that extending the time of starch to act with lipid resulted in higher content of starch-lipid complex. While the amount of oil was further increased to 15 mL, the RS content decreased from 0.61 to 0.20 g/100 g when the stir-frying time increased from 30 to 120 s. At this condition, the noodles were similar to fried noodles, and the RS content was similar to that of the fried noodles.

After frying, only a small amount of RS (0.47 g/100 g) remained in the noodles when the oil temperature was 140 °C; it decreased significantly (0.25 g/100 g) as the frying time increased. There was no RS be detected by using the assay kit when the frying temperature was above 160 °C (Figure 1e, Table S5).

Comparing the optimal cooking conditions of the five different cooking methods, noodles cooked using microwave showed the highest RS content (0.99 g/100 g), followed by the stir-fried noodles (0.59 g/100 g). The RS contents of the steamed and boiled noodles were similar, 0.44 and 0.43 g/100 g, respectively. With the same cooking time and temperature (180 °C for 30 s), the RS content of stir-fired noodles was ranged between 0.52–0.61 g/100 g, while no RS was detected in the fried noodles (Table 1).

### 3.2. Effects of Different Cooking Methods on the in Vitro Starch Digestibility and Estimated Glycemic Index of Noodles

**Figure 2:**
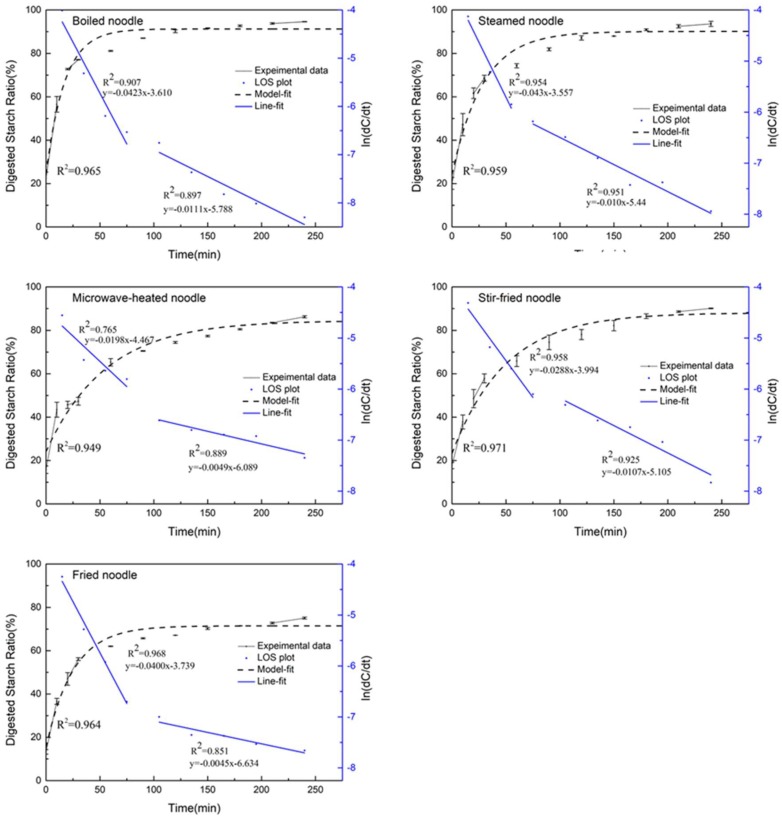
Starch digestion curves, model-fit curves and Logarithm of Slope (LOS) plots from cooked noodles starch.

The digestive properties of optimally cooked noodles were investigated. The LOS plot was used in this study to quantify the differences in the digestion rate (*k*) and the extent of starch digested (*C_∞_*) during starch amylolysis [25]. Digestgrams of the starch hydrolysis of cooked noodles are shown in Figure 2. All the digestion of starch within noodles can be divided into two stages, with a faster digestion rate in the first stage, and a much slower digestion rate in the second stage. The starch was digested rapidly during the initial one hour and nearly reached a plateau at 60–80 min (Figure 2), with a rate constant ranging between 0.0411 and 0.0202 min^−1^. The two digestion phases were similar to the results of Yao [9]. The *k*_1_ value is more physiologically relevant, as the food digestion in the first 120 min can reflect the release rate of glucose after a meal and is given more importance compared to *k*_2_. Besides, there were no significant differences in the second stage digestion rates (*k*_2_) among the noodles cooked using different methods. Thus, we mainly focused on the changes in the *k*_1_, which showed the main differences in the digestive rates among different noodles.

The boiled and fried noodles were digested quicker (*k*_1_ value of 0.0411 and 0.0423 min^−1^, respectively) than noodles prepared using other cooking methods, which was followed by steamed noodles (0.0354 min^−1^). The digestion rates of noodles after microwave cooking and stir-frying were slower, 0.0202 and 0.0290 min^−1^, respectively. 

The maximum hydrolysis extent, *C_∞_*, ranged between 77.48% (frying) and 96.22% (boiling). Among the five cooking methods, the steamed and boiled noodles showed the largest digestibility (96.22% and 94.22%, respectively). The digestibility of starch in stir-fried noodles was slightly lower (90.46%), which is similar to the study of Reed [28]. The digestibility of noodles after frying and microwave was significantly lower.

The values of estimated glycemic index (eGI) of noodles cooked using different methods are shown in Table 2. However, the eGI value of noodles cooked using different methods varied greatly because the eGI was calculated using the *k* and *C_∞_*. The eGI value of noodles followed the order, boiled noodle (98.33) > steamed noodle (95.55) > stir-fried noodle (90.46) > microwave cooked noodle (86.93) > fried noodle (86.18). Although there were some differences in the eGI of the noodles obtained by the five cooking methods, they were still among the high GI food (GI ≥ 75).

### 3.3. Thermal Transitions and Crystalline Structure of Starch in Noodles Cooked using Different Methods

**Figure 3:**
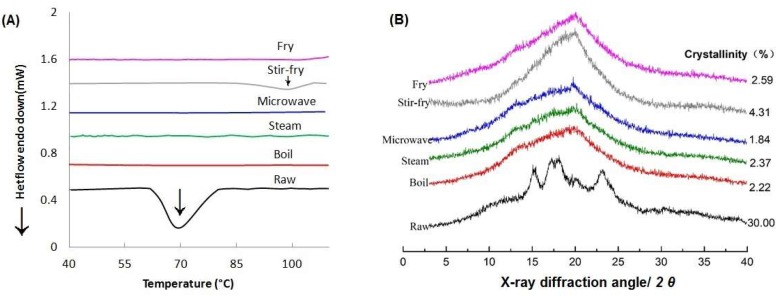
The thermal transition (**A**) and X-ray polymorphs (**B**) of differently-cooked noodles.

The peak gelatinization temperature of raw wheat starch was 69.61 °C (Figure 3 and Table 1). This is slightly higher than the value reported (62.4 °C) [29], which might be related to the variety and origin of wheat starch. Compared with raw starch, the endothermic peak position (at about 70°) of starch within noodles disappeared after cooking (Figure 3A), indicating the starch granules within noodles were fully gelatinized after cooking under the optimum cooking conditions. 

The crystalline polymorph of raw wheat starch in the uncooked noodles was of the type-A crystal (Figure 3B), with the degree of crystallinity about 30%. The distinctive peaks of A-type polymorph disappeared after cooking, whereas peaks (around 8° and 20°) were observed in all the cooked noodles, which was the characteristic peak of V-type polymorph [30]. The V-type crystals may be formed by the amylose and lipid within wheat flour. The degree of crystallinity of noodles cooked using different methods at the optimum cooking condition ranged from 1.84% to 4.31%, and it was highest in the noodles after being stir-fried (4.31%) (Figure 3B).

Notably, an endotherm peak was observed in the stir-fried noodles, with a peak shown at around 98 ℃, which is similar to the study of Yang et al. [27]. This endotherm peak suggesting that starch-lipid complexes formed during stir-frying [31,32]. As indicated by Ai et al. [33], the phase transition temperature of RS_5_ (amylose-lipid complex) was ranged from 98.6 to 99.8 °C. The Δ*H* value of stir-fried noodles (0.84 J/g) was similar to the result of Yang [27], that the Δ*H* of the starch-lipid complexes of the steamed-fried noodles ranged from 1.01 to 1.40 J/g. However, this peak was not found in the fried noodles. There was a pre-boiling step in the preparation of stir-fried noodles, which enabled water (Table 1) to be absorbed and to facilitate amylose-lipid complex formation [34]. Lack of water of fried noodle compared with stir-fried noodles may explain no such complex observed.

### 3.4. The Morphology of Noodles Cooked Using Different Methods

**Figure 4:**
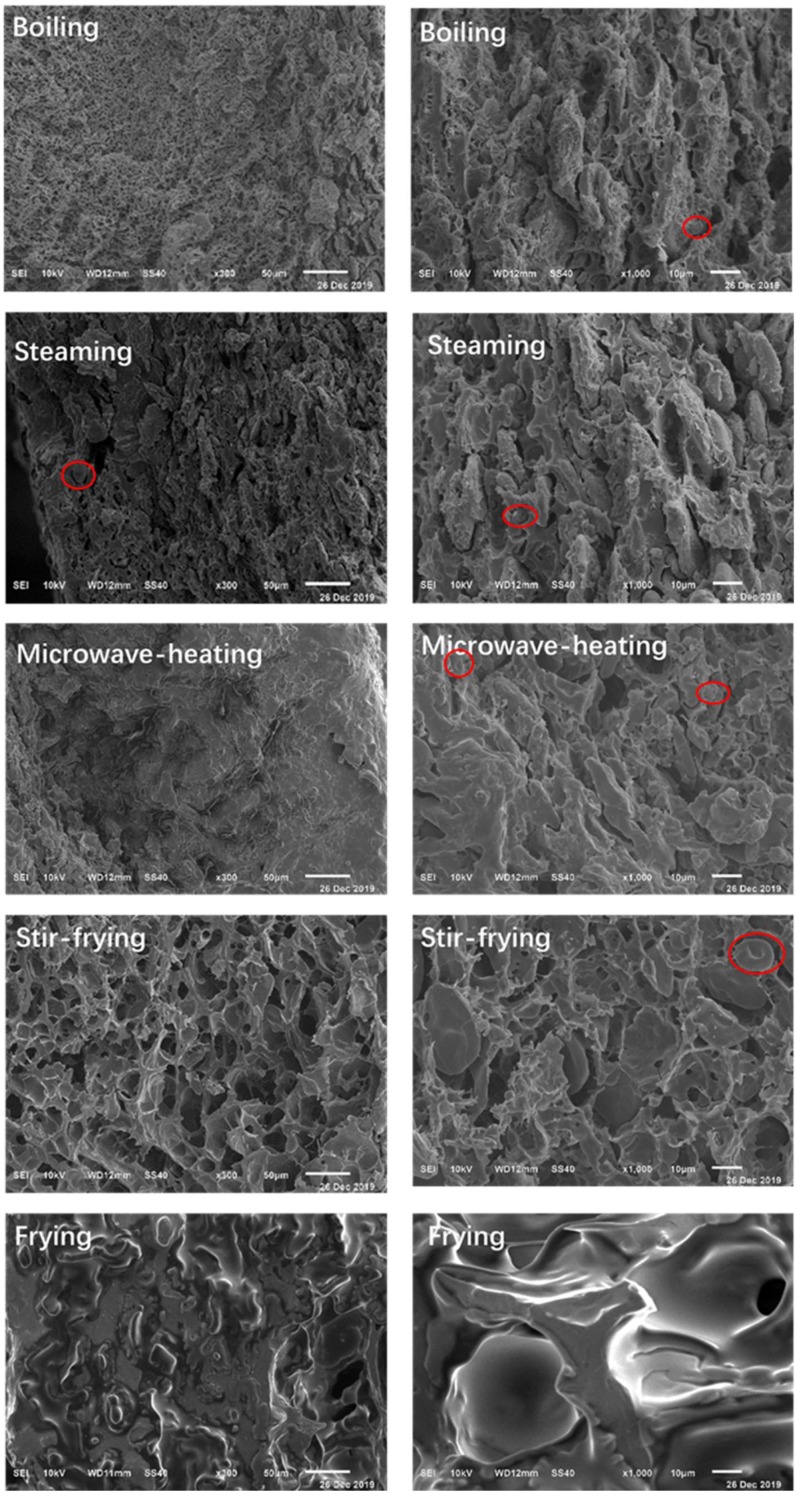
The morphology of noodles cooked using different methods (Left: Edge structure ×300; Right: Center structure ×1000).

The morphology of noodles cooked using different methods were obviously different regarding the outer layer and inner structures. As shown in Figure 4, the edge of noodles showed dense pore structure after boiling with a fine sponge-like shape. Relatively large voids also appeared at the edge of the steamed noodles compared to the boiled noodles; however, more starch granules (red circle) were embedded in the protein network structure within the steamed noodles. Compared with the boiled and steamed noodles, the noodles after microwave showed almost no voids on the edges. 

**Figure 5:**
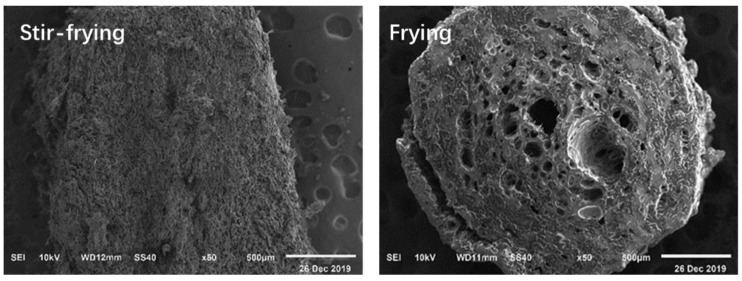
The morphology of stir-fried and fried noodles (×50).

For the stir-fried and fried cooking methods, the noodles treated at a higher temperature showed significantly different morphology than other noodles, i.e., starch was cracked and deformed to varying degrees, and holes of different sizes were produced in the stir-fried and fried noodles (Figure 5). For instance, the stir-fried noodles showed uniform and dense pores, while the fried noodles showed some larger holes and were irregularly distributed, similar to previous studies [35,36]. Stir-fried noodles were honeycomb-shaped and contain more porous cavities (Figure 4), with some starch and gluten protein stuck together. The surface of the fried noodles was uneven and the surface protein of the fried noodles was difficult to restrain the expanded starch particles.

For the inner structure, the starch granules in the noodles cooked using different methods were swelled to different degrees. The inner structure was denser than the external structure (Figure 4), which was similar to the results of Sekine [37]. The internal structure of steamed noodles was similar to that of boiled noodles, that the starch granules were destroyed, forming starch aggregates with fragmented surfaces. Due to the rapid rise in temperature during microwave heating, the starch granules within microwaved noodles swelled to a lower degree with a dense network structure formed by the starch and protein.

From the morphology changes of noodles cooked using different methods, the order of structure destruction of noodles can be boiling > steaming > microwaving. The stir-fried and fried noodles also have significantly different internal structures. Deeper and larger holes were formed inside the fried noodles. This was due to a large amount of water evaporated during the frying process [38]. Stir-fried noodles showed higher moisture content, relatively smaller holes, and less starch damage compared to fried noodles.

## 4. Discussion 

The RS content and digestibility of the noodles cooked using different methods were varied by the different cooking methods. It is noted that there was still a small amount of RS within cooked noodles when they were cooked to the OCT (Table 1). A small portion of the starch coated with protein or fat was still present in the cooked noodles, forming RS_1_. During the cooking process, the water migrated from the surface layer of the noodles to the center. Although starch of the noodles had been mostly gelatinized, the degree of gelatinization of the starch on the surface of the noodles was higher than the internal [37] (Figure 4), and there might be a small amount of ungelatinized starch (RS_1_) inside the noodles. On the other hand, from the results of XRD (Figure 3B), diffraction peaks at different degrees of noodles cooked using different methods appeared around 2*θ* of 20°, indicated that the starch and lipid in the noodles after boiling and steaming form a partial complex, namely RS_5_.

Although boiled and steamed noodles showed similar RS contents (Table 1), their digestion rates were significantly different (Table 2). The boiled noodles were cooked in excess water, while the steamed noodles were cooked without direct contact with water. From the SEM results, the morphology of the steamed noodles showed less porous surface than the boiled noodles (Figure 4), leading to a slower digestion rate of the steamed noodles than that of the boiled noodles.

The microwaved noodles showed lower digestion rate and higher RS content than the boiled or steamed noodles due to the less swelled starch granules. Heating with microwave irradiation is caused by molecular friction resulting from the dielectric coupling of molecules [39], which resulted in a faster heating rate than in conductively heated samples. Different from the findings on the pure starch under microwave cooking [40] in excess water, the starch granules within microwaved noodles were swelled in a limited degree and there was not porous or holes on the edges of the noodles (Figure 4). This was also different from the conventional cooked (boiled and steamed) noodles. The pores on the surface of starch granules will facilitate the digestive enzymes to access the starch [41], which can influence the digestive properties. It was reported the gelatinization of microwaved starch granules (in a short time) required more energy than the conductive heated samples [42], which might be related to the higher RS content of microwaved noodles as which showed a more compact structure; however, this correlation between the swelling degree and digestion rate needs to be further verified. 

For stir-fried and fried noodles, starch can form amylose-lipid compounds (RS_5_) easily [43], as the noodles may react more with oil during the cooking process. The variance in the RS contents of stir-fried and fried noodles might be explained by their different moisture contents, as water is a necessary plasticizer during the formation of the complex [44]. The RS content of stir-fried noodles was much higher than that of the fried noodles (Table 1). The moisture content of the stir-fried noodles (52.42%) was higher than that of the fried noodles (0.67%), as there was a pre-cooking step for the stir-fried noodles. With 52.42% moisture content, the starch granules were more likely to form the starch-lipid complex (Figure 3). The absence of water in the deep-fried noodles greatly reduced the formation of amylose-lipid complex, leading to almost no RS in the fried noodles. Singh et al. [45] also found that fried and baked potatoes exhibit less content of RS than the raw and shallow fried potatoes when the water availability was limited. Moreover, there were more and larger holes present in the fried noodles, while the noodles after stir-fried were exhibited smaller holes and relatively dense structure (Figure 4 and Figure 5). As a result, more RS_5_ was formed in the stir-fried noodles and its digestion rate was lower.

The RS content of noodles had a significant negative correlation with its digestion rate (Table 3), that is, the noodles with higher RS content had a slower digestion rate. However, the trend of RS and *C_∞_* was not consistent. For example, there was no RS within the fried noodles but the extent of starch digested was 77.48%, which was the lowest among cooked noodles. The reason for the difference between the RS content and the final digestibility may be due to the methods for measuring the RS content of in vitro digestion, which is mainly different in the duration of digestion. The test method for RS content using kit lasted for 16 hours, while it only lasted 5 hours for the in vitro digestion method. Similar difference in the RS content and *C_∞_* was also observed by Hasjim [46], but it merits further investigations on the difference of the residue structures and its fermentation properties with the same microorganisms. 

According to the K-RSTAR 06/18 Megazyme kit, higher errors can be obtained for samples with RS contents < 2%. The repeatability to measure the RS content was justified using some of the cooked noodles, where the standard deviation was between 0.01 and 0.12 (g/100 g). Thus, although the RS content was low in some cooked noodles, the value can still be compared in this investigation.

## 5. Conclusions

Cooking conditions affect the amount of RS in noodles, and the RS content of the noodles was not changed when further extended the cooking time when the optimal cooking time is reached. The RS content in noodles after microwave cooking and stir-frying was highest, while there was no RS in the fried noodles. Moisture content may influence the RS_5_ formation during noodle cooking, since more RS_5_ was formed in stir-fried noodles. The eGI value of the noodles obtained using different cooking methods was also different, which indicates that it is not accurate to estimate the GI value of the type of food using single value, as the eGI can be varied by the way of cooking. Besides the cooking methods, food storage conditions may also impact the structure and digestive properties of starch-based foods differently, which merits further investigation. In addition, the digestive properties of different cooked noodles in this study were analyzed through in vitro experiments, whether similar results can be obtained through in vivo analysis requires further experiments.

## Figures and Tables

**Table 1:** RS content and moisture content of raw noodle and cooked noodles (cooked under optimum cooking time (OCT)).

Cooking Method	Moisture Content/%	*T_o_* (°C)	*T_p_* (°C)	*T_c_* (°C)	△*H* (J/g)	RS Content/%
Raw	12.79 ± 0.35 ^e^	63.13 ± 0.65 ^b^	69.61 ± 0.08 ^b^	80.69 ± 0.86 ^c^	4.55 ± 0.98 ^a^	13.83 ± 0.12 ^a^
Boiling	67.69 ± 0.17 ^a^	-	-	-	-	0.43 ± 0.01 ^c^
Steaming	55.50 ± 1.28 ^c^	86.05 ± 1.41 ^a^	99.10 ± 0.08 ^a^	109.84 ± 0.01 ^a^	0.38 ± 0.08 ^b^	0.44 ± 0.01 ^c^
Microwave heating	63.68 ± 1.52 ^b^	-	-	-	-	0.99 ± 0.05 ^a^
Stir-frying	52.42 ± 0.79 ^c^	88.39 ± 4.54 ^a^	97.74 ± 0.82 ^a^	104.20 ± 2.74 ^b^	0.84 ± 0.95 ^b^	0.59 ± 0.01 ^c^
Frying	0.67 ± 0.03 ^d^	-	-	-	-	0 ^d^

Values are means ± standard deviation. Values with different letters in the same column are significantly different at *p* < 0.05.

**Table 2:** The digestion rate and digestibility of cooked noodles.

Cooking Methods	*k*_1_(min^−1^)	*k*_2_(min^−1^)	*C_∞_* (%)	HI	eGI
Boiling	0.0411 ± 0.002 ^a^	0.0110 ± 0.0002 ^a^	96.22 ± 0.76 ^a^	106.78 ± 0.53 ^a^	98.33 ± 0.29 ^a^
Steaming	0.0354 ± 0.002 ^b^	0.0111 ± 0.0009 ^a^	94.22 ± 1.42 ^a^	101.71 ± 0.19 ^a^	95.55 ± 0.11 ^a^
Microwave-heating	0.0202 ± 0.000 ^c^	0.0054 ± 0.0004 ^b^	88.71 ± 0.67 ^c^	86.01 ± 1.57 ^c^	86.93 ± 0.86 ^c^
Stir-frying	0.0290 ± 0.001 ^d^	0.0127 ± 0.0020 ^a^	91.53 ± 0.35 ^b^	92.43 ± 2.80 ^b^	90.46 ± 1.54 ^b^
Frying	0.0423 ± 0.003 ^a^	0.0041 ± 0.0004 ^b^	77.48 ± 0.50 ^d^	84.64 ± 1.36 ^c^	86.18 ± 0.75 ^c^

*k*_1_, the digestion rate of starch in the first stage; *k*_2_, the digestion rate of starch in the second stage; *C_∞_*, the theoretical digestibility of starch digestion endpoint; HI, hydrolysis index; eGI, estimated glycemic index. Values are means ± standard deviation. Values with different letters in the same column are significantly different at *p* < 0.05.

**Table 3:** Spearman correlation coefficients among RS content and digestion properties.

	RS Content
*C_∞_*	0.012
*k*_1_	−0.924 **

** indicates an extremely significant correlation between them (*p* < 0.01). *k*_1_ value represents the digestion rate of starch in the first stage; *k*_2_ value represents the digestion rate of starch in the second stage; *C_∞_* represents the theoretical digestibility of starch digestion endpoint.

## Supplementary Materials

The following are available online at https://www.mdpi.com/2304-8158/9/3/328/s1, Table S1: Effect of different boiling time on the RS content, Table S2: Effect of different steaming time on RS content, Table S3: Effect of microwave time on RS content, Table S4: Effect of adding oil and stir-frying time on RS content, Table S5: Effect of oil temperature and frying time on RS content.
