# Supplementary material for: RS Content and eGI Value of Cooked Noodles (I): Effect of Cooking Methods
Source: Foods. 2020 Mar 11;9(3):328. doi: 10.3390/foods9030328 (PMC7143191; doi:10.3390/foods9030328)
Supplement: Supplementary file 1 [file foods-09-00328-s001.pdf]

Table S1. Effect of different boiling time on the RS content

Boiling Time/min	RS content(g/100g)
2	0.60±0.01a
2.5	0.49±0.01b
3	0.44±0.01c
3.5	0.43±0.01c
4	0.41±0.01c

Table S2. Effect of different steaming time on RS content

Steaming time/min	RS content(g/100g)
10	0.79±0.01a
15	0.44±0.00b
20	0.39±0.03c
30	0.38±0.02c

Table S3. Effect of microwave time on RS content

Microwave heating time（min）	RS content(g/100g)
60s	1.36±0.12a
120s	1.0±0.04b
150s	0.99±0.03b
180s	1.01±0.03b
240s	0.95±0.03b

Table S4. Effect of adding oil and stir-frying time on RS content

**Stir-frying conditions (oil, time)**	**RS content(g/100g)**
5 mL 30s	0.52±0.01d
5 mL 120s	0.90±0.01a
10 mL 30s	0.59±0.01c
10 mL 120s	0.76±0.01b
15 mL 30s	0.61±0.01c
15 mL 120s	0.20±0.01e

Table S5. Effect of oil temperature and frying time on RS content

**Frying conditions (temperature, time)**	**RS content(g/100g)**
140℃ 30s	0.47±0.02a
140℃ 120s	0.25±0.00b
160℃ 30s	0c
160℃ 120s	0c
180℃ 30s	0c
180℃ 120s	0c
200℃ 30s	0c
200℃ 120s	0c
